# The complete chloroplast genome of *Clematis henryi var. ternata* (Ranunculaceae)

**DOI:** 10.1080/23802359.2021.1907807

**Published:** 2021-03-31

**Authors:** Xiang Chen, Qinxiang Chang, Pengguo Xia, Zongsuo Liang, Kaijing Yan

**Affiliations:** a Key Laboratory of Plant Secondary Metabolism and Regulation of Zhejiang Province, College of Life Sciences and Medicine, Zhejiang Sci-Tech University, Hangzhou, China; b Taiyuan University, Taiyuan, China; c Tasly Pharmaceutical Group Co., Ltd, Tianjin, China

**Keywords:** Chloroplast genome, *Clematis henryi var.ternata*, phylogenetic analyses

## Abstract

The complete chloroplast genome of *Clematis henryi var.ternata* was determined in this study. The genome was 159,675 base pair (bp) in length, containing a large single-copy (LSC) region of 79,443 bp, a small single-copy region (SSC) of 18,100 bp and a pair of inverted repeats (IRs) of 31,066 bp. It contains 130 unique genes, including 86 protein-coding genes, 36 transfer RNA (tRNA) genes, and eight ribosomal RNA (rRNA) genes. The GC content of the complete chloroplast genome sequence was 38.0%. Phylogenetic analyses using complete chloroplast genomes showed that *Clematis henryi var.ternata* is most closely related to *Clematis guniuensis* (NC_050373.1).

There were about 300 species in the genus *Clematis* L. It is distributed on all continents, mainly in tropical and subtropical regions, as well as in cold regions (Tamura 1995; Johnson 1997; Wang and Li 2005). There were about 108 species in China, which were distributed throughout the country, especially in the southwest (Wang and Li 2005). Among them, *Clematis henryi var.ternata* is produced in the southwestern of Shannxi province, China. In this study, we analyzed the characters of the complete chloroplast genome sequence for *Clematis henryi var.ternate* to confirm its phylogenetic position and evolutionary relationship between the *Clematis henryi var.ternata* and other Ranunculaceae species.

*Clematis henryi* has the functions of promoting qi and relieving pain, promoting blood circulation and reducing swelling. As a traditional herbal medicine, *Clematis henryi* has been widely used in China for hundreds of years for the treatment of infectious and inflammatory disorders. Pharmacological studies have shown that *Clematis henryi* has obvious analgesic and sedative effects, and has analgesic effects on head, stomach, abdominal muscles and joint pain (Liu 2007). In this article, we de novo assembled the complete chloroplast genome of *Clematis henryi var.ternata*, which will provide genomic data for the related studies and a phylogenetic tree was generated to reveal its relationship with other *Clematis* species.

The samples of *Clematis henryi var.ternata* were collected from Ningshan County, Shannxi Province, China (33°32′28.01″N, 108°37′39.59″E). The voucher specimen was preserved at XBGH (The Herbarium of Xi’an Botanical Garden) (Voucher number: *Xun Lulu* et al.*00361*). Total genomic DNA was extracted using the modified CTAB method (Doyle and Doyle 1987) and TruSeq DNA Sample Prep Kit (Illumina, USA) was used to construct a genomic library consisting of an insert size of 350 bp. Sequencing was carried out on an Illumina NovaSeq 6000 platform. Raw PE reads of about 2.23 Gb were trimmed and high quality PE reads of about 2.21 Gb were subjected to de novo assembly. The NOVOplasty (Dierckxsens et al. 2017) were used for the de novo assembly of chloroplast genome. The sequence was annotated using GeSeq (https://chlorobox.mpimp-golm.mpg.de/geseq-app.html), and visually checked in Geneious v8.0.2 (Kearse et al. 2012) using the chloroplast genome of *Clematis guniuensis* (GenBank accession number (QMS50675.1)) as reference. Finally, a complete chloroplast genome of *Clematis henryi var.ternata* was obtained and submitted to Genbank (GenBank accession number (MW380948)).

The complete chloroplast genome of *Clematis henryi var.ternata* (GenBank accession number: (MW380948)) was 159,675 bp in length with 38.0% GC contents, consisting of a pair of inverted repeat regions (IRa and IRb) with same length (31,066 bp) separated by the large single copy (LSC, 79,443 bp) and small single copy (SSC, 18,100 bp) regions. A total of 130 genes were identified, including 86 protein-coding genes, eight ribosomal RNA (rRNA) genes, and 36 transfer RNA (tRNA)genes.

Phylogenetic tree was constructed using IQTREE v1.6.7(Nguyen et al. 2015) for *Clematis henryi var.ternata* and 41 Ranunculaceae species chloroplast genomes, with the best selected TVM + F+R4 model and 5000 bootstrap replicates. The chloroplast genome of the *Clematis henryi var.ternata* showed the closest relationship to that of previously reported *Clematis guniuensis* (NCBI reference sequence ID NC_050373.1, 159,682 bp in length) (Jiang et al. 2020) (Figure 1). The newly characterized *Clematis henryi var. ternata* complete chloroplast genome would provide essential data for further study on the phylogeny and evolution of *Clematis* L. and provide useful resources for better understanding the physiology and evolution of Ranunculaceae. The phylogenetic tree illuminates the phylogenetic relationships among *Clematis* species.

**Figure 1.**
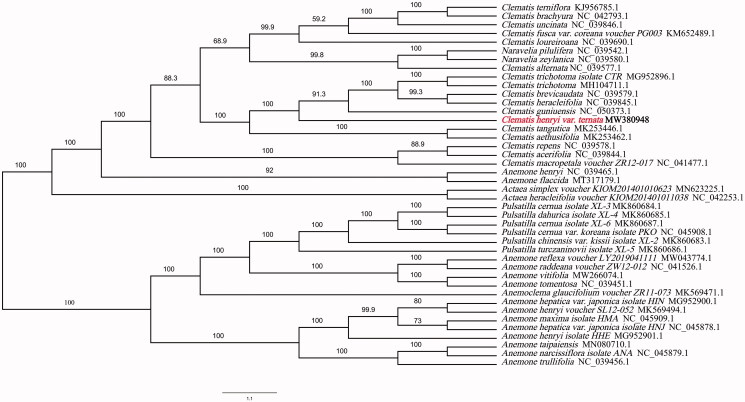
Phylogenetic tree showing the relationship between *Clematis henryi var.ternata* and 41 Ranunculaceae species. Phylogenetic tree was constructed based on the complete chloroplast genomes using maximum likelihood (ML) with 5000 bootstrap replicates. Numbers in each the node indicated the bootstrap support values.

## Data Availability

The data that support the findings of this study are openly available in NCBI (https://www.ncbi.nlm.nih.gov) GenBank with the accession number (MW380948).
